# Prevalence and characteristics of *rmtB* and *qepA* in *Escherichia coli* isolated from diseased animals in China

**DOI:** 10.3389/fmicb.2013.00198

**Published:** 2013-07-15

**Authors:** Yu-Ting Deng, Zhen-Ling Zeng, Wei Tian, Tong Yang, Jian-Hua Liu

**Affiliations:** ^1^National Reference Laboratory of Veterinary Drug Residues, College of Veterinary Medicine, South China Agricultural UniversityGuangzhou, China; ^2^Key Laboratory of Fishery Drug Development, Ministry of Agriculture, P. R. China, Pearl River Fisheries Research Institute, Chinese Academy of Fishery ScienceGuangzhou, China

**Keywords:** 16S rRNA methylases, *qepA*, *E. coli*, animal, molecular typing

## Abstract

16S rRNA methylase and QepA, a fluoroquinolone efflux pump, are new mechanisms of resistance against aminoglycosides and fluoroquinolone, respectively. One of 16S rRNA methylase genes, *rmtB*, was found to be associated with *qepA*, were both located on the same transposable element. In this study, we intended to determine the current prevalence and characteristics of the 16S rRNA methylase genes and *qepA*, and to study the association between *rmtB* and *qepA*. A total of 892 *Escherichia coli* isolates were collected from various diseased food-producing animals in China from 2004 to 2008 and screened by PCR for 16S rRNA methylase genes and *qepA*. About 12.6% (112/892) and 0.1% (1/892) of isolates that were highly resistant to amikacin were positive for *rmtB* and *armA*, respectively. The remaining five 16S rRNA methlyase genes were not detected. Thirty-six (4.0%) strains carried *qepA*. About 32.1% of *rmtB*-positive strains harbored *qepA*, which was not detected in *rmtB*-negative strains. Most strains were clonally unrelated, while identical PFGE profiles of *rmtB*-positive isolates were found in the same farm indicating clonal transmission. Conjugation experiments showed that *rmtB* was transferred to the recipients, and *qepA* also cotransferred with *rmtB* in some cases. The spread of *E. coli* of food animal origin harboring both *rmtB* and *qepA* suggests that surveillance for antimicrobial resistance of animal origin as well as the study of the mechanisms of resistance should be undertaken.

## Introduction

In China, aminoglycosides and quinolone were commonly used for treating severe infections caused by Gram-negative bacteria in animal husbandry. As a result, multiple resistance determinants to these antimicrobial agents have emerged in various pathogenic microbes. Recently, a new type of mechanism, post-transcriptional methylation of the 16S rRNA, has been reported, and this results in high-level resistance to aminoglycoside antibiotics. At present, ten 16S rRNA methylase genes have been identified, including *armA, rmtA, rmtB, rmtC, rmtD, rmtE, rmtF, rmtG, rmtH*, and *npmA*, which are capable of conferring extraordinarily high levels of resistance to most clinically important aminoglycosides, including amikacin, gentamicin, kanamycin, and tobramycin (Galimand et al., 2003; Yokoyama et al., 2003; Doi and Arakawa, 2007; Wachino et al., 2007; Davis et al., 2010; Galimand et al., 2012; Bueno et al., 2013; O'Hara et al., 2013). RmtB and ArmA are the most frequently identified methylases in *Enterobacteriaceae* isolated in East Asia, Europe, and South America (Yan et al., 2004; Bogaerts et al., 2007; Berçot et al., 2008; Kang et al., 2009; Yu et al., 2010). The plasmid mediated efflux pump gene, *qepA*, which confers resistance to hydrophilic fluoroquinolones by efflux, has also been reported recently to be frequently associated with the *rmtB* gene (Périchon et al., 2007; Yamane et al., 2007; Deng et al., 2011b; Yao et al., 2011).

The emergence of 16S rRNA methylases in bacteria of animal origin was first discovered in *E. coli* isolates of pig origin harboring the *armA* gene in Spain in 2005 (González-Zorn et al., 2005). Since then, 16S rRNA methylase-producing *E. coli* isolates have been increasingly detected in pigs, chicken, cows, and companion animals (dogs and cats) in different countries (Chen et al., 2007; Liu et al., 2008; Du et al., 2009; Davis et al., 2010; Hopkins et al., 2010; Deng et al., 2011a). Stains from food-producing animals colonized with 16S rRNA methylase conferring high level of resistance to aminoglycosides have been considered a potential source of resistant *E. coli* causing infection in the community (Chen et al., 2007). A combination of factors has contributed to the rapid dissemination of 16S rRNA methylase genes, such as co-selection, which is mainly due to conjugative plasmids and other mobile genetic elements rather than clonal expansion. Some studies have reported that the dissemination of the *rmtB* gene involves IS*26*, Tn3, Tn1721, IS*CR1*, and IS*CR3* (Yamane et al., 2007; Berçot et al., 2008; Périchon et al., 2008; Du et al., 2009). In this study, we intended to investigate the distribution of 16S rRNA methylase genes and *qepA* among *E. coli* isolates originating from various food-producing animals in China from 2004 to 2008. In addition, in order to understand how the *rmtB* and *qepA* genes disseminated, molecular typing and conjugation experiments were conducted to determine the mechanisms of resistance and mobility of the *rmtB* and *qepA* genes in these isolates.

## Materials and methods

### Bacterial isolates

*E. coli* isolates were recovered from feces, livers, lungs, or milk samples of diseased food-producing animals with diarrhea, respiratory diseases or other diseases, including 360 pigs, 261 chickens, 179 ducks, 34 geese, 9 pigeons, 12 partridges, and 37 cows in six provinces of China from July 2004 to October 2008. Each isolate was from a separate animal, and a total of 892 isolates were collected from 150 farms. All samples were seeded on MacConkey agar plates and incubated at 37°C for 24 h. The isolates were identified with conventional biochemical tests and confirmed using an API 20E system (bioMérieux, Marcy l'Étoile, France). *E. coli* ATCC25922 was used as the MIC reference strain and *E. coli* J53 Azide^R^ as the recipient for conjugation experiments.

### Detection of 16S rRNA methylases genes and qepA

A multiplex-PCR method was used to detect the genes enconcoding 16S rRNA methylases (*armA, rmtA, rmtB, rmtC, rmtD*) with primer sets described previously (Doi and Arakawa, 2007). The primers used to amplify *npmA* were 5′-AGG GCT ATC TAA TGT GGT G-3′ and 5′-TAT TTC CGC TTC TTC GTA T-3′. The *qepA* gene was amplified with primers 5′-GCA GGT CCA GCA GCG GGT AG-3′ and 5′-CTT CCT GCC CGA GTA TCG TG-3′. The PCR products were confirmed by sequencing.

### Antimicrobial susceptibility testing

The minimal inhibitory concentrations (MICs) of ampicillin, ceftiofur, nalidixic acid, ciprofloxacin, norfloxacin, gentamicin, amikacin, streptomycin, neomycin, apramycin, chloramphenicol, florfenicol, and sulfamethoxazole/trimethoprim were determined by the agar dilution method according to Clinical and Laboratory Standards Institute (CLSI) guidelines (CLSI, 2008, 2010). *E. coli* ATCC25922 was used as a quality control strain.

### Molecular typing

Pulsed-field gel electrophoresis (PFGE) analysis of *Xba*l-digested genomic DNA was performed to determine the genetic relatedness of 16S rRNA methylase-producing *E. coli* isolates using a CHEF-II System (Bio-Rad Laboratories, Hercules, CA, USA) with PFGE separation conditions of 0.5–63.8 s for 20.3 h. PFGE patterns were interpreted according to well established criteria (Tenover et al., 1995). Isolates that had PFGE patterns with no more than six different bands were considered to be clonally related.

All 16S rRNA methylase-positive isolates were classified into phylogenetic groups (A, B1, B2, or D) according to the presence of *chuA, yjaA*, and TSPE4, as determined by an established multiplex PCR-based method described previously (Clermont et al., 2000).

### Conjugation experiment

In order to test the transferability of the amikacin resistance determinant to the azide-resistant strain J53, the *rmtB*-positive stains were employed as putative donors in a conjugation assay using the broth mating method. The transconjugants were selected on LB agar plates supplemented with amikacin (200 μg/mL) and sodium azide (200 μg/mL). From the transconjugants, PCRs for *rmtB* and *qepA* were performed using primers reported previously. The MICs for donors, transconjugants, and recipients were measured by the agar dilution method in accordance with CLSI guidelines. The antimicrobials tested were amikacin, gentamicin, nalidixic acid, ciprofloxacin, norfloxacin, ampicillin and other antimicrobials.

## Results

### Detection of 16S rRNA methylases genes and qepA

All isolates, which were derived from various diseased food-producing animals, were screened for 16S rRNA methylase genes by PCR. One *armA* and 112 *rmtB* genes were found to be present in these isolates, representing 0.1 and 12.6% of the total 892 *E. coli* isolates, respectively. None of other five 16S rRNA methylase genes were detected in any of the isolates. As shown in Table 1, the distribution of 16S rRNA methylase genes in different animal species was varied. Details of *armA* and *rmtB*-positive isolates were listed in Table A1.

**Table 1:** **Distribution of the 16S rRNA methylase genes and *qepA* among all isolates from different sources**.

**Resistance gene**	**No. of isolates (%)**						**No. of PFGE subtypes**
	**Pigeon and partridge**	**Goose and duck**	**Cow**	**Chicken**	**Pig**	**Total**	
	**(*n* = 21)**	**(*n* = 213)**	**(*n* = 37)**	**(*n* = 261)**	**(*n* = 360)**	**(*n* = 892)**	
*armA*	–	1	–	–	–	1	1
*rmtB*	6	27	4	10	29	76	58, N/T(10)^a^
*rmtB, qepA*	7	9	–	10	10	36	21, N/T(12)
Sum of *rmtB*	13 (61.9)	36 (16.9)	4 (10.8)	20 (7.7)	39 (10.83)	112 (12.6)	79, N/T(22)

PFGE, pulsed field gel electrophoresis; N/T, non-typeable.

a Number of non-typeable isolates was indicated in parentheses.

The *qepA* gene was detected in 36 of 892 isolates (4.0%). About 32.1% of *rmtB*-positive strains harbored *qepA*, while *qepA* was not detected in *rmtB*-negative strains.

### Susceptibility testing results

The susceptibility testing to 14 antimicrobial agents was conducted for the 113 isolates found to be 16S rRNA methylase-positive. All the strains producing 16S rRNA methylases displayed high-level resistance (MIC > 128 μg/ml) to amikacin and gentamicin, as well as to ampicillin, tetracycline, streptomycin, and nalidixic acid. In addition, there was also a very high frequency of resistance to sulfamethoxazole/trimethoprim, florfenicol, and chloramphenicol among these isolates: 97.2, 93.7, and 92.4%, respectively. Of the 113 isolates, all were multi-resistant and demonstrated resistance to ciprofloxacin (88.6%), norfloxacin (82.3%), neomycin (75.9%), apramycin (59.5%), and ceftiofur (42.3%) (Table 2).

**Table 2:** **Susceptibility of 113 *E. coli* isolates carrying 16S rRNA methylases to 14 antimicrobial agents**.

**Antimicrobial agents**	**Susceptible (%)**	**Resistant (%)**	**MIC range (μg/ml)**	**MIC_50_ (μg/ml)**	**MIC_90_ (μg/ml)**
Amikacin	0	100	>1024	>1024	>1024
Gentamicin	0	100	512- > 1024	>1024	>1024
Nalidixic acid	0	100	>128	>128	>128
Ampicillin	0	100	>128	>128	>128
Tetracycline	0	100	32- > 128	>128	>128
Streptomycin	0	100	32- > 128	>128	>128
Florenicol	1.3	93.7	2- > 128	>128	>128
Trimethoprim/Sulfamethoxazole	2.8	97.2	1/19 >64/1216	>64/1216	>64/1216
Chloramphenicol	5.1	92.4	4- > 128	>128	>128
Ciprofloxacin	8.9	88.6	0.125- > 64	>64	>64
Norfloxacin	10.1	82.3	0.125- > 128	>128	>128
Neomycin	16.5	75.9	1- > 128	>128	>128
Apramycin	16.5	59.5	2- > 128	16	>128
Ceftiofur	53.8	42.3	0.06- > 128	4	>128

### Molecular typing

In order to characterize the clonality of all 16S rRNA methylase-positive strains, molecular typing was performed by PFGE analysis and phylogenetic group assignment was carried out. PFGE was performed on 113 16S rRNA methylase-producers, and 91 isolates were found to be typeable while 22 were non-typeable. Seventy-nine major profiles were obtained among the 90 *rmtB*-positive strains, and 60 of them were represented by a single isolate (Table 3).

**Table 3:** **Distribution of 16S rRNA methylase- positive strains, their clonal relationship, and phylogenetic groups from different animal species**.

**Animal species**	**No. of 16S rRNA methylases-positive strains/farms**	**No. of PFGE subtypes**	**No. of phylogenetic groups (A/B1/B2/D)**
Pigeon and partridge	13/3	7, N/T(6)^a^	9/1/0/3
Goose and duck	37/15	24, N/T(10)	26/6/0/5
Chicken	20/12	15, N/T(4)	11/4/1/4
Cow	4/2	3	1/2/0/1
Pig	39/22	30, N/T(2)	32/7/0/0
Total	113/54	79, N/T(22)	79/20/1/13

PFGE, pulsed field gel electrophoresis; N/T, non-typeable.

a Number of non-typeable isolates was indicated in parentheses.

The majority of *armA*- and *rmtB*-producing isolates were found to belong to phylogenetic group A (*N* = 79, 69.9%). Twenty (17.7%), and 13 (11.5%) of the strains were found to belong to groups B1 and D, respectively. Only one *rmtB*-positive strain from chicken belonged to group B2.

### Conjugation experiment

Plasmid transfer of high-level aminoglycoside resistance to *E. coli* J53 was successful for 43 of the 65 *rmtB*-carrying strains. The *qepA* gene also co-transferred with the *rmtB* gene from 17 *rmtB*-*qepA*-positive donor strains, and its presence was confirmed in all of 17 transconjugants by PCR. The MICs of amikacin and gentamicin for all *rmtB*-carrying transconjugants were 256- to 512-fold higher than those for the recipients, indicating that the *rmtB* gene contributes to high-level resistance to aminoglycosides. Furthermore, the plasmids in some transconjugants also confered resistance to ampicillin (100%), tetracycline (6.8%), chloramphenicol (25.0%), nalidixic acid (9.12%), trimethoprim/sulfamethoxazole (40.9%) and ceftiofur (29.5%). Compared to the *qepA*-negative transconjugants, which displayed no changes in susceptibility to fluoroquinolone, the MICs of ciprofloxacin and norfloxacin for *qepA*-harboring transconjugants were 8- to 64-fold higher than those found for the recipients. These results suggest that *qepA* contributes to the decrease in hydrophilic fluoroquinolone susceptibility.

## Discussion

### Detection of 16S rRNA methylases genes and qepA

In this study, 892 *E. coli* isolates originating from various diseased food-producing animals, including 360 pigs, 261 chickens, 179 ducks, 34 geese, 9 pigeons, 12 partridges, and 37 cows, were collected from six different provinces in China from 2004 to 2008. We found that 12.6% (112/892) of them carried *rmtB* gene, while the *armA* gene was detected in only one isolate. This result demonstrates the widespread dissemination of the *rmtB* gene among multiple animal sources in China, as reported in other studies (Chen et al., 2007; Liu et al., 2008; Du et al., 2009; Deng et al., 2011a; Li et al., 2012). The prevalence rate of *rmtB* among *E. coli* isolated from different animals was much higher than those reported to be of patient origin, which was less than 5% (Fritsche et al., 2008; Kang et al., 2008). Nonetheless, the incidence of *armA*-carrying *Enterobacteriaceae* isolates of patient origin is higher in comparison with the incidences determined from data obtained regarding isolates derived from animal sources (Lee et al., 2006; Kang et al., 2008).

QepA is a plasmid-mediated efflux pump first discovered in an *E. coli* strain isolated from the urine specimen of an inpatient in Japan in 2002 (Yamane et al., 2007). The plasmid bearing the *qepA* gene increased the MICs of nalidixic acid, ciprofloxacin and norfloxacin by 2-, 32-, and 64-fold, respectively. Since its initial discovery, a variant of *qepA* possessing two amino acid substitutions was identified and named QepA2 (Cattoir et al., 2008); this variant conferred a resistance phenotype similar to that of QepA, which has now been renamed QepA1. Interestingly, *qepA1*-positive isolates from Japan, Belgium, China, and South Korea were found to be associated with the *rmtB* gene on the same Tn3 transposon (Périchon et al., 2007; Yamane et al., 2007; Périchon et al., 2008; Kim et al., 2009; Deng et al., 2011b), whereas *qepA2* was flanked by a novel element, IS*CR3C*, with no *rmtB* associated with it (Cattoir et al., 2008). In this study, the *qepA* gene was found in 36 of 892 isolates (4.0%), and 32.1% of *rmtB*-positive strains harbored *qepA*. *qepA* was not detected in *rmtB*-negative strains, implying that *qepA* gene was associated with *rmtB* gene on a same mobile genetic element as reported recently (Liu et al., 2008; Périchon et al., 2008; Yamane et al., 2008; Park et al., 2009; Yao et al., 2011), except a recent report by Baudry et al. (2009). The high prevalence of *rmtB* and *qepA* coexisting in *E. coli* samples of diseased food-producing animals is worrisome, because they may be rapidly spreading among animals, humans and even in the environment; this may be occurring by direct or indirect contact and co-selection, with various antimicrobials possibly contributing to its dissemination and further limiting therapeutic options.

### Molecular typing of the 16S rRNA methylases-positive strains

Among the 112 *rmtB*-positive strains, 79 distinct PFGE patterns were found, and 21 isolates concomitantly harbored *qepA* gene. Only small numbers of isolates with *rmtB* were clonally related. PFGE analysis indicated that a diversity of PFGE patterns was present in strains of different origins. However, identical patterns were found in the strains derived from the same farm. These data suggested that the high prevalence of *rmtB*-positive isolates was not mainly caused by clonal dissemination. Phylogenetic background of the 113 16S rRNA methylases-positive strains were also conducted in this study. Studies have shown that phylogenetic groups B2 and D usually carry virulence factors (Clermont et al., 2000). Compared with the other phylogenetic groups, a greater number of isolates in phylogenetic group B2 from human patients has been reported (Baudry et al., 2009; Song et al., 2009). However, we only obtained one *rmtB*-positive strain from chicken belonged to B2. The strains collected from diseased animals belong mainly to group A, including *armA*-positive, *rmtB*-positive and *qepA*-positive strains, indicating that most strains were not involved in pathogenicity.

### Conjugation experiment of 16S rRNA methylases-positive strains

To investigate the transferability of *rmtB* to the recipient *Ecoli* J53, conjugation experiment was conducted. Forty three *rmtB*-positive conjugative plasmids were obtained, 17 of which carried both *rmtB* and *qepA*. *E. coli* isolates harbored transferable aminoglycoside resistance determinants and the increasing prevalence of transferable quinolone resistance determinants may have been an important driving force for selection and dissemination of aminoglycoside- and quinolone-resistant isolates. Our previous work showed that *rmtB* and *qepA* were found located on a very similar F2:A-:B- plasmids, which have disseminated among pigs, human and environment (Deng et al., 2011b). Another reports showed that 24 out of 35 transconjugants bearing *rmtB* and *qepA* originating from companion animals were also associated with the F2:A-:B- plasmid, 21 of which were sharing the identical plasmid restriction patterns (Deng et al., 2011a). It suggested that co-existence of *rmtB* and *qepA* on the same plasmid may contribute to dissemination of both aminoglycoside and quinolone resistance in different animal sources.

In conclusion, the present screen revealed a high prevalence of 16S rRNA methylase genes among *E. coli* isolated from various diseased food-producing animals in six provinces of China from 2004 to 2008. The dissemination of *rmtB* and *qepA* genes in the *E. coli* of food-producing animals was mainly mediated by a conjugated plasmid. The coexistence of these resistance determinants on a single plasmid increases the selection by one or more of the antimicrobials used in clinical practice. Prudent use of antimicrobial agents in veterinary clinics, especially those treating food-producing animals, should be reinforced.

### Conflict of interest statement

The authors declare that the research was conducted in the absence of any commercial or financial relationships that could be construed as a potential conflict of interest.

## Appendix

**Table A1:** **Details of *armA* and *rmtB*-positive isolates**.

**Isolates^a^**	**Province^b^**	**Animal species**	**Sample sources**	**Year**	**Resistance genes^c^**	**Phylogenetic type**	**Resistance phenotype^d^**
GDD31	GD	duck	liver	2007	*armA*	A	CHL, FFC, NOR, CIP, SXT, TET
BJC01, BJC02	BJ	chicken	unknown	2004	*rmtB*	A, B1	(FFC, NOR, CIP), CHL, SXT, TET
GDC05, GDC04, GDC01	GD	chicken	liver	2004	*rmtB*	A, B1	CHL, FFC, SXT, NEO, TET
GDC07, GDC08	GD	chicken	feces	2005	*rmtB*	A	CHL, FFC, NOR, CIP, SXT, TET
FJC01, FJC02	FJ	chicken	liver	2007	*rmtB*	A	CHL, FFC, NOR, CIP, SXT, TET
GDC06, GDC01	GD	chicken	liver	2007	*rmtB*	D	CHL, FFC, NOR, CIP, SXT, NEO, TET
GDN01, GDN02, GDN03, GDN04	GD	cow	milk	2007	*rmtB*	B1, A, D	(CTF, APR), CHL, FFC, NOR, CIP, TET
GDD01	GD	duck	unknown	2004	*rmtB*	A	CHL, FFC, NOR, CIP, SXT, NEO, TET
GDD25	GD	duck	feces	2004	*rmtB*	D	CHL, FFC, NOR, CIP, SXT, CTF, NEO, APR, TET
GDD32,GDD12, GDD16,GDD17, GDD20, GDD02, GDD06, GDD04, GDD33, GDD30, GDD28, GDD29, GDD11, GDD19	GD	duck	liver	2007	*rmtB*	A, B1, D	(CHL, FFC, NOR, CIP, SXT, CTF, NEO, APR), TET
GDD13, GDD14, GDD115, GDD22, GDD08	GD	duck	feces	2007	*rmtB*	A, B1	(CTF, APR), CHL, FFC, NOR, CIP, SXT, TET
GDD03, GDD09	GD	duck	liver	2008	*rmtB*	D	(CHL, FFC, SXT,CTF), NOR, CIP, NEO, TET
GDD24	GD	duck	feces	2008	*rmtB*	A	CHL, FFC, NOR, CIP, SXT, CTF, NEO, APR, TET
GDE02	GD	geese	liver	2007	*rmtB*	A	CHL, FFC, NOR, CIP, SXT, NEO, TET
GDE03, GDE04	GD	geese	feces	2007	*rmtB*	D, A	(NEO), CHL, FFC, SXT, APR, TET
GDZ01, GDZ02, GDZ05	GD	partridge	liver	2005	*rmtB*	D, A	(CTF, APR), CHL, FFC, NOR, CIP, SXT, NEO, TET
GDZ07	GD	partridge	feces	2005	*rmtB*	A	CHL, FFC, NOR, CIP, SXT, CTF, NEO, APR, TET
GDP29, GDP30, GDP31, GDP25, GDP24, GDP26, GDP27, GDP32, GDP34	GD	pig	unknown	2004	*rmtB*	A, B1	CHL, FFC, NOR, CIP, SXT, NEO, TET
GDP03	GD	pig	unknown	2005	*rmtB*	A	CHL, FFC, NOR, CIP, SXT, TET
GDP05	GD	pig	lung	2007	*rmtB*	A	CHL, FFC, NOR, CIP, SXT, APR, TET
GDP11,GDP36, GDP06	GD	pig	feces	2007	*rmtB*	A	(FFC, NOR, CTF, APR), CHL, CIP, SXT, NEO, TET
GDP22,GDP10, GDP09, GDP19, GDP20, GDP21, GDP23, GDP35	GD	pig	liver	2007	*rmtB*	A, B1	(CHL, FFC, CIP, NEO), NOR, SXT, APR, TET
JXP01	JX	pig	liver	2007	*rmtB*	A	CHL, FFC, NOR, CIP, SXT, NEO, APR, TET
GDP07,GDP16, GDP15, GDP08	GD	pig	sneeze	2008	*rmtB*	B1, A	(CHL, FFC, NOR, CIP, NEO), SXT, TET
GDP17, GEP13	GD	pig	liver	2008	*rmtB*	A	(CTF, APR), CHL, FFC, NOR, CIP, SXT, NEO, TET
GDP18	GD	pig	kidney	2008	*rmtB*	B1	CHL, FFC, NOR, CIP, SXT, NEO, APR,TET
GDG01, GDG02	GD	pigeon	feces	2007	*rmtB*	A, B1	(CTF), CHL, FFC, NOR, CIP, SXT, APR, TET
GDG03	GD	pigeon	liver	2008	*rmtB*	A	CHL, FFC, NOR, CIP, SXT, CTF, APR, TET
BJC03, BJC04, BJC05	BJ	chicken	unknown	2004	*rmtB, qepA*	D, A	CHL, FFC, NOR, CIP, SXT, NEO, TET
GDC02, GDC03, GDC09	GD	chicken	feces	2004	*rmtB, qepA*	A, B1	CHL, FFC, NOR, CIP, SXT, NEO, TET
HNC04, HNC01, HNC02, HNC03	HN	chicken	unknown	2004	*rmtB, qepA*	D, B2, A	CHL, FFC, NOR, CIP, SXT, NEO, TET
GDD27, GDD26	GD	duck	feces	2004	*rmtB, qepA*	A, B1	CHL, FFC, NOR, CIP, SXT, NEO, APR, TET
GDD05, GDD10, GDD18, GDD21	GD	duck	liver	2007	*rmtB, qepA*	A	CHL, FFC, NOR, CIP, SXT, CTF, NEO, APR, TET
GDD07	GD	duck	feces	2007	*rmtB, qepA*	A	CHL, FFC, NOR, CIP, SXT, NEO, TET
GDD23	GD	duck	sneeze	2008	*rmtB, qepA*	A	CHL, FFC, NOR, CIP, SXT, CTF, NEO, TET
GDE01	GD	geese	liver	2005	*rmtB, qepA*	A	CHL, FFC, NOR, CIP, NEO, APR, TET
GDZ04,GDZ03	GD	partridge	liver	2005	*rmtB, qepA*	A	CHL, FFC, NOR, CIP, SXT, NEO, APR, TET
GDZ06,GDZ10, GDZ09, GDZ08	GD	partridge	feces	2005	*rmtB, qepA*	A, D	CHL, FFC, NOR, CIP, SXT, NEO, APR, TET
GDP01, GDP02, GDP28	GD	pig	unknown	2004	*rmtB, qepA*	A, B1	(FFC, CTF, NEO), CHL, NOR, CIP, SXT, TET
SCP01	SC	pig	unknown	2004	*rmtB, qepA*	A	CHL, FFC, NOR, CIP, SXT, TET
GDP33	GD	pig	lung	2007	*rmtB, qepA*	A	CHL, FFC, SXT, NEO, TET
GDP04, GDP12	GD	pig	liver	2007	*rmtB, qepA*	A	CHL, NOR, CIP, SXT, CTF, NEO, APR, TET
GDP37	GD	pig	feces	2007	*rmtB, qepA*	A	CHL, FFC, NOR, CIP, SXT, TET
GDP14	GD	pig	sneeze	2008	*rmtB, qepA*	A	CHL, FFC, NOR, CIP, SXT, NEO, TET

a Isolates sharing the same number were obtained from the same sample. Isolates with transconjugants were underlined.

b GD, Guangdong; BJ, Beijing; FJ, Fujian; JX, Jiangxi; HN, Henan; SC, Sichuan.

c Genes that were transferred by conjugation as determined by PCR were underlined.

d All isolates and transconjugants were resistant to gentamicin, amikacin and amplicin. All isolates were also resistant to nalidixic acid, tetracycline and streptomycin. CHL, chloramphenicol; FFC, florfenicol; NOR, norfloxacin; CIP, ciprofloxacin; SXT, trimehoprim/ sulfamethoxazole; CTF, ceftiofur; NEO, neomycin; APR, apramycin; TET, tetracycline. Resistance to antimicrobial agents appearing in parentheses was not present in all isolates.
